# ﻿Discovery of the males of Lasioglossum (Eickwortia)hienae and L. (E.) alexanderi, with new distributional records for the species (Apoidea, Halictidae)

**DOI:** 10.3897/zookeys.1228.136718

**Published:** 2025-02-14

**Authors:** Ismael A. Hinojosa-Díaz, Ana Celeste Martínez Cervantes, Jason Gibbs

**Affiliations:** 1 Departamento de Zoología, Instituto de Biología, Universidad Nacional Autónoma de México (UNAM), Tercer Circuito s/n, Ciudad Universitaria, Coyoacán, A.P. 70-153, Ciudad de México, C.P. 04510, Mexico; 2 Posgrado en Ciencias Biológicas, Universidad Nacional Autónoma de México, Unidad de Posgrado, Circuito de Posgrados, Edificio D, Ciudad Universitaria, Coyoacán, Ciudad de México, C.P. 04510, Mexico; 3 Department of Entomology, University of Manitoba, 12 Dafoe Road, Winnipeg, MB, R3T 2N2, Canada

**Keywords:** Costa Rica, description, identification key, Mexico, taxonomy

## Abstract

Within the diverse genus *Lasioglossum*, the subgenus Eickwortia as currently understood, encompasses three characteristic species from mountainous areas in Mexico and Central America. Prior to this work only Lasioglossum (Eickwortia) nycteris had descriptions for both male and female. Here we describe and illustrate the previously unknown males of L. (E.) hienae and L. (E.) alexanderi, providing new distributional data for both species, and an updated species identification key for the group.

## ﻿Introduction

The bee genus *Lasioglossum* Curtis, 1833 stands out in terms of species richness and morphological challenges to discriminate groups of species within it (Michener 2007; Gibbs et al. 2013). While the over 1800 species (Ascher and Pickering 2024) in the genus can be assigned to two informal series, *Lasioglossum* series and *Hemihalictus* series (Danforth et al. 2003; Michener 2007), the subgeneric classification is still in flux (Gibbs et al 2012; Gibbs 2018; Gardner 2023). The *Hemihalictus* series is characterized by the weakening or loss of vein 1rs-m of the forewing. The subgenus Eickwortia is a Neotropical member of the *Hemihalictus* series (Michener 2007; Gibbs and Dumesh 2013; Gardner 2023), originally described as a separate genus (McGinley 1999) for two species, *L.nycteris* (Vachal, 1904) and *L.alexanderi* (McGinley, 1999), the latter known only from the holotype, which is currently lost (Gibbs and Dumesh 2013). Both species occur in mountainous areas in Mexico and Central America. A third species, *L.hienae* Gibbs & Dumesh, 2013, was described based on a single female from the state of Colima, Mexico. Lasioglossum (Eickwortia) as conceived for the inclusion of the three mentioned species can be characterized by black integument, infuscate wings, and females with noticeably bidentate mandibles (McGinley 1999; Gibbs and Dumesh 2013). Only the male of *L.nycteris* has been described and is characterized by a slender metasoma, reminiscent of *Neocorynura*, and a slender hind basitarsus (McGinley 1999).

Recent phylogenetic evidence based on molecular data (Gardner 2023) shows that *Eickwortia* (*sensu stricto*) is nested within a clade of metallic, Neotropical species leading to an expanded definition of the subgenus, *Eickwortia* (*sensu lato*). This larger clade includes the so called *Augochlora*-like *Dialictus* (Gardner 2023), a distinctive group of species defined by Engel et al. (2007) as the “*aurora* species group”. These metallic *Eickwortia* have generally been treated as Lasioglossum (Dialictus), although their position outside of L. (Dialictus) has been understood for some time (Danforth et al. 2003; Gibbs et al. 2012, 2013). Here we provide the description for the previously unknown males of Lasioglossum (Eickwortia) hienae and L. (E.) alexanderi, with new distributional records for the species.

## ﻿Material and methods

Material examined for this study corresponded to 44 specimens, including the five newly described males. The Lasioglossum (Eickwortia) hienae specimens are all held in Mexican institutions: Colección Nacional de Insectos, Instituto de Biología, Universidad Nacional Autónoma de México (CNIN) (1 ♂, 3 ♀♀); Colección Himenopterológica, Museo de Zoología “Alfonso L. Herrera”, Facultad de Ciencias, Universidad Nacional Autónoma de México (MZFC) (2 ♂, 21 ♀♀); Colección de la Estación de Biología Chamela, Instituto de Biología, Universidad Nacional Autónoma de México (EBCh) (3 ♀♀). Lasioglossum (E.) nycteris males were examined from the Snow Entomological Collection, Lawrence, Kansas, USA (2 ♂♂) and Colección Nacional de Insectos, Instituto de Biología, Universidad Nacional Autónoma de México (CNIN) (2 ♂♂). The new L. (E.) alexanderi records are deposited in the J.B. Wallis / R.E. Roughley Museum of Entomology (WRME), University of Manitoba, Winnipeg, Canada (2 ♂♂, 7 ♀♀) and Bee Biology and Systematics Laboratory (BBSL), Logan, Utah, USA (1 ♀).

The new males were associated to the female by the strong morphological similarity and the co-occurrence of both sexes during the same collecting events. Males of *L.hienae* can be identified as L. (Eickwortia) based on their remarkable similarity to *L.nycteris*. Males of *L.alexanderi* were collected with the female, which was readily identified using the description and photographs in McGinley (1999).

Morphological terminology for the male descriptions follows that of McGinley (1999) for the male of L. (E.) nycteris (the only *Eickwortia* male known before this work), as well as the description provided by Gibbs and Dumesh (2013) for the female of L. (E.) hienae. The interpretation of some characters is based on other sources (Michener and Fraser 1978; Brooks 1988; Michener 2007). Surface sculpture follows Harris (1979) terminology. Length measurements for L. (E.) hienae are presented as the average of the three available male specimens, showing individual lower and higher measurements in parenthesis, while for L. (E.) alexanderi meassurements for the two available specimens are shown. Length ratios are presented as the average for the measured specimens. Lengths of body setae are referred as comparison to ocellar diameter (OD). The genitalia were dissected from one male *L.hienae* and cleared in a solution of sodium hydroxide (NaOH) at room temperature for 12 hours. Photomicrographs were produced through a Zeiss Axio Zoom V16 microscope with an Axiocam MRC5 camera or a Canon EOS 7D camera with MPE-65 lens mounted on a copy stand with a StackShot system.

## ﻿Results

### ﻿ Lasioglossum (Eickwortia) hienae

Taxon classificationAnimaliaHymenopteraHalictidae

Gibbs & Dumesh

B8600B34-130B-58BC-AD51-EED4F9133EB1

Figs 1–5 , 6–8

Lasioglossum (Eickwortia) hienae Gibbs and Dumesh 2013: 3 (♀).

#### Material examined.

30 specimens (3 ♂♂ previously unknown, 27 ♀♀). Mexico • 1 ♂; Oaxaca, Putla Villa de Guerrero, Entre Asunción Atoyaquillo y Cerro Campana; 16.71075°N, 97.80907°W; 1730 m a.s.l.; 07 Nov. 2015; I. Hinojosa/ C. Martínez leg.; Red aérea; OAX165; CNIN • 1 ♂; Oaxaca, Pluma Hidalgo, Ranchería San Isidro; 15.94056°N, 96.40083°W; 1255 m a.s.l.; 19 Sep. 2007; Omar Ávalos Hernández leg.; ejemplar_id: 1088 catálogo: 31964; OAH -215 [pencil/handwritten]; MUSEO DE ZOOLOGIA HYMENOPTERA 31964; MZFC • 1 ♂; Oaxaca, Pluma Hidalgo, Cascada mayor; 15°55'11"N, 96°23'34"W; 900 m a.s.l.; 19 Sep. 2007; E. Suaste leg.; ES01; MUSEO DE ZOOLOGIA HYMENOPTERA, 31873; 9–10 am; MZFC • 1 ♀; Oaxaca, Putla Villa de Guerrero, Entre Asunción Atoyaquillo y Cerro Campana; 16.71075°N, 97.80907°W; 1730 m a.s.l.; 07-Nov-2015; Red aérea; Col. I. Hinojosa, C. Martínez leg.; OAX162; CNIN • 1 ♀; Oaxaca, Putla Villa de Guerrero, Entre Asunción Atoyaquillo y Cerro Campana; 16.71075°N, 97.80907°W; 1730 m a.s.l.; 07-Nov-2015; Red aérea; Col. I. Hinojosa, C. Martínez leg.; OAX163; CNIN • 1 ♀; Oaxaca, Putla Villa de Guerrero, Entre Asunción Atoyaquillo y Cerro Campana; 16.71075°N, 97.80907°W; 1730 m a.s.l.; 07-Nov-2015; Red aérea; Col. I. Hinojosa, C. Martínez leg.; OAX164; CNIN • 1 ♀; Oaxaca, Pluma Hidalgo, Ranchería San Isidro; 15.94056°N, 96.40083°W; 1255 m a.s.l.; 19 Sep. 2007; Omar Ávalos Hernández leg.; ejemplar_id: 1089 catálogo: 31965; OAH -215 [pencil/handwritten]; MUSEO DE ZOOLOGIA HYMENOPTERA 31965; MZFC • 1 ♀; Oaxaca, Pluma Hidalgo, Ranchería San Isidro; 15.94056°N, 96.40083°W; 19 Sep. 2007; Andrés Ressendiz Flores leg.; ejemplar_id: 1080 catálogo: 31956; AR -03 [pencil/handwritten]; MUSEO DE ZOOLOGIA HYMENOPTERA 31956; MZFC • 1 ♀; Oaxaca, Pluma Hidalgo, Ranchería San Isidro; 15.94056°N, 96.40083°W; 19 Sep. 2007; Marycarmen Jiménez de Loera leg.; ejemplar_id: 1100 catálogo: 31976; MJ 04 [pencil/handwritten]; MUSEO DE ZOOLOGIA HYMENOPTERA 31976; MZFC • 1 ♀; Oaxaca, Pluma Hidalgo, Ranchería San Isidro; 15.94056°N, 96.40083°W; 19 Sep. 2007; Marycarmen Jiménez de Loera leg.; ejemplar_id: 1105 catálogo: 31981; MJ 06 [pencil/handwritten]; MUSEO DE ZOOLOGIA HYMENOPTERA 31981; MZFC • 1 ♀; Oaxaca, Pluma Hidalgo, Ranchería San Isidro; 15.94056°N, 96.40083°W; 19 Sep. 2007; Marycarmen Jiménez de Loera leg.; ejemplar_id: 1108 catálogo: 31984; MJ 06 [pencil/handwritten]; MUSEO DE ZOOLOGIA HYMENOPTERA 31984; MZFC • 1 ♀; Oaxaca, Pluma Hidalgo, Ranchería San Isidro; 15.94056°N, 96.40083°W; 1255 m a.s.l.; 19 Sep. 2007; Marycarmen Jiménez de Loera leg.; ejemplar_id: 1113 catálogo: 31989; MJ 10 [pencil/handwritten]; MUSEO DE ZOOLOGIA HYMENOPTERA 31989; MZFC • 1 ♀; Oaxaca, Pluma Hidalgo, Ranchería San Isidro; 15.94056°N, 96.40083°W; 1255 m a.s.l.; 19 Sep. 2007; Marycarmen Jiménez de Loera leg.; ejemplar_id: 1114 catálogo: 31990; MJ 10 [pencil/handwritten]; MUSEO DE ZOOLOGIA HYMENOPTERA 31990; MZFC • 1 ♀; Oaxaca, Pluma Hidalgo, La Soledad; 15°58'18"N, 96°31'54"W; 1550 m a.s.l.; 21 Sep. 2007; Marycarmen Jiménez de Loera leg.; ejemplar_id: 1119 catálogo: 31995; MJ 15 [pencil/handwritten]; MUSEO DE ZOOLOGIA HYMENOPTERA 31995; 09:30 h; MZFC • 1 ♀; Oaxaca, La Soledad; 15°58'18"N, 96°31'54"W; 1550 m a.s.l.; 19 Sep. 2007; E. Suaste leg.; ES07; MUSEO DE ZOOLOGIA HYMENOPTERA 32002; 11:30 am-12:30 pm; MZFC • 1 ♀; Oaxaca, Rancho San Isidro; 15.94056°N, 96.40083°W; 1255 m a.s.l.; 19 Sep. 2007; Y. Ramírez leg.; YR07; MUSEO DE ZOOLOGIA HYMENOPTERA 29804; 13:00–14:00 hrs; MZFC • 1 ♀; Oaxaca, La Soledad; 15°58'18"N, 96°31'54"W; 1550 m a.s.l.; 21 Sep. 2007; Y. Ramírez leg.; YR10; MUSEO DE ZOOLOGIA HYMENOPTERA 29801; 9:00–10:00 am; MZFC • 1 ♀; Oaxaca, La Soledad; 15°58'18"N, 96°31'54"W; 1550 m a.s.l.; 21 Sep. 2007; Y. Ramírez leg.; YR10; MUSEO DE ZOOLOGIA HYMENOPTERA 29802; 9:00–10:00 am; MZFC • 1 ♀; Oaxaca, La Soledad; 15°58'18"N, 96°31'54"W; 1550 m a.s.l.; 21 Sep. 2007; Y. Ramírez leg.; YR10; MUSEO DE ZOOLOGIA HYMENOPTERA 29803; 9:00–10:00 am; MZFC • 1 ♀; Oaxaca, La Soledad; 15°58'18"N, 96°31'54"W; 1550 m a.s.l.; 21 Sep. 2007; Y. Ramírez leg.; YR11; MUSEO DE ZOOLOGIA HYMENOPTERA 29805; 10:00–11:00 am; MZFC • 1 ♀; Oaxaca, La Soledad; 15°58'18"N, 96°31'54"W; 1550 m a.s.l.; 21 Sep. 2007; Y. Ramírez leg.; YR11; MUSEO DE ZOOLOGIA HYMENOPTERA 29806; 10:00–11:00 am; MZFC • 1 ♀; Oaxaca, La Soledad; 15°58'18"N, 96°31'54"W; 1550 m a.s.l.; 19 Sep. 2007; E. Suaste leg.; ES05; MUSEO DE ZOOLOGIA HYMENOPTERA 31814; 9:30–10:30 am; MZFC • 1 ♀; Oaxaca, Pluma Hidalgo- Ranchería San Isidro “cafetal”; 15.94056°N, 96.40083°W; 1255 m a.s.l.; 19 Sep. 2007; A. Díaz leg.; DIMA03; MUSEO DE ZOOLOGIA HYMENOPTERA 31854; 9:03 am; MZFC • 1 ♀; Oaxaca, Pluma Hidalgo, Cascada mayor; 15°55'11"N, 96°23'34"W; 900 m a.s.l.; 19 Sep. 2007; E. Suaste leg.; ES01; MUSEO DE ZOOLOGIA HYMENOPTERA, 31868; 9–10 am; MZFC • 1 ♀; Oaxaca, Pluma Hidalgo, Cascada mayor; 15°55'11"N, 96°23'34"W; 900 m a.s.l.; 19 Sep. 2007; E. Suaste leg.; ES01; MUSEO DE ZOOLOGIA HYMENOPTERA, 31870; 9–10 am; MZFC • 1 ♀; Oaxaca, Pluma Hidalgo, Cascada mayor; 15°55'11"N, 96°23'34"W; 900 m a.s.l.; 19 Sep. 2007; E. Suaste leg.; ES01; MUSEO DE ZOOLOGIA HYMENOPTERA, 31871; 9–10 am; MZFC • 1 ♀; Oaxaca, Pluma Hidalgo, Cascada mayor; 15°55'11"N, 96°23'34"W; 900 m a.s.l.; 19 Sep. 2007; E. Suaste leg.; ES01; MUSEO DE ZOOLOGIA HYMENOPTERA, 31872; 9–10 am; MZFC.• 1 ♀; Colima, Hacienda San Antonio; 19.45192°N, 103.71394°W; 1190 m a.s.l.; 26 Oct. 2015; R. Ayala leg.; a15; EBCH • **1** ♀; Colima, Hacienda San Antonio; 19.45192°N, 103.71394°W; 1190 m a.s.l.; 26 Oct. 2015; R. Ayala leg.; a16; EBCH • 1 ♀; Colima, Hacienda San Antonio; 19.45192°N, 103.71394°W; 1190 m a.s.l.; 26 Oct. 2015; R. Ayala leg.; EBCH.

**Figures 1–5.**
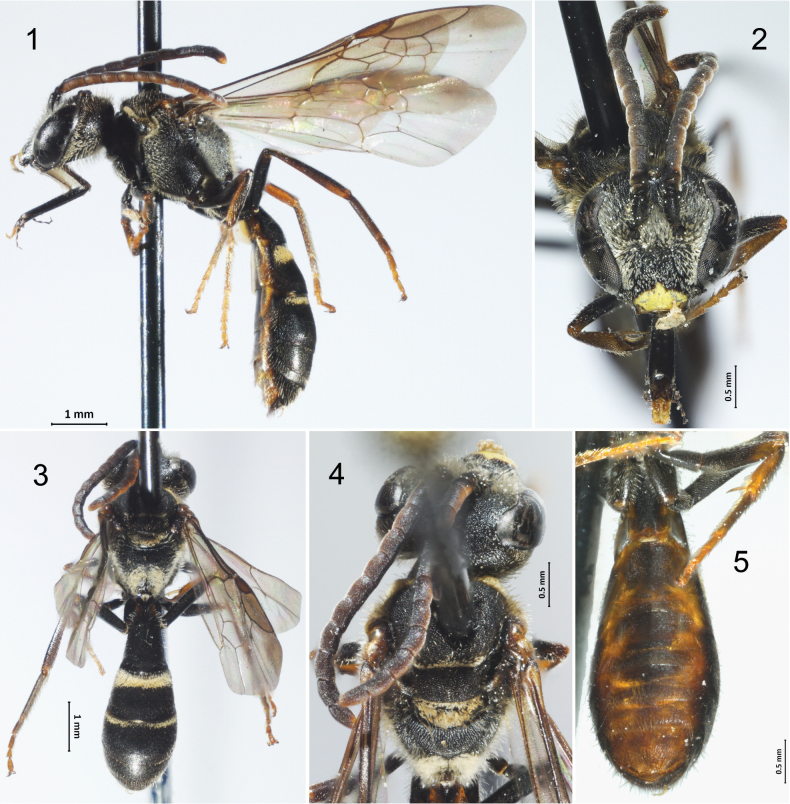
Male of Lasioglossum (Eickwortia) hienae**1** lateral habitus **2** face **3** dorsal habitus **4** dorsal view of mesosoma **5** ventral view of metasoma.

**Figures 6–8.**
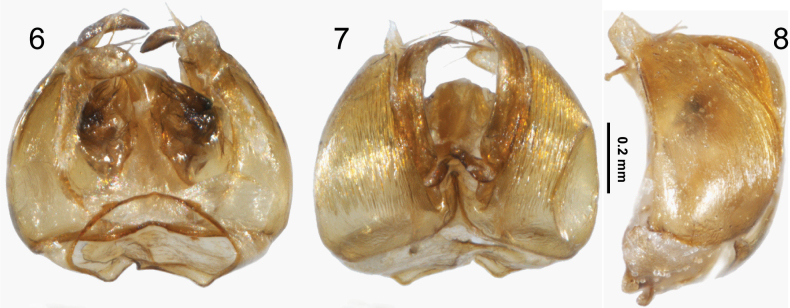
Male genitalic capsule of Lasioglossum (Eickwortia) hienae**6** ventral aspect **7** dorsal aspect **8** lateral aspect.

#### Diagnosis.

Males of *L.hienae* resemble male *Neocorynura* due to the slender T1, but they can be distinguished by the characteristics of the tribe Halictini, which include the reflexed apex of T7, with a subapical ridge creating a false apex. Male *L.hienae* can be recognized by their black integument, yellow clypeal mark, anteriorly infuscate wings, and distinct tomentum on the metanotum, posterior propodeum, and base of T2. There is currently no reliable way to distinguish the males of *L.hienae* and *L.nycteris*.

Both species share unique characters within male *Lasioglossum* in the Western Hemisphere: inner metatibial spur short or absent entirely, T1 narrow (distinctly longer than wide), and hind legs long and slender.

#### Description.

**Male**: Length 8.39 mm (8.14–8.62 mm).

Head length 2.03 mm (1.91–2.17 mm). Head width 2.13 mm (2.05–2.19 mm). Intertegular span 1.47 mm (1.39–1.58 mm).

Forewing length (Total) 6.53 mm (6.34–6.76 mm), forewing length (M-2rm) 2.39 mm (2.28–2.55 mm).

Maximum metasomal width (T3) 1.64 mm (1.58–1.72 mm).

***Structure*.** Head approximately as long as wide (length/width ratio = 0.95). Gena width at midlength 0.74 × width of contiguous section of compound eye in lateral view. Supraclypeal area weakly protuberant. Clypeus width approximately twice length, width/length ratio = 2.04, projecting approximately 0.65 its length below lower margin of eyes; clypeal protuberance 0.26 mm (0.21–0.32 mm).

Ocellocular distance 2.60 × lateral ocellar diameter, approximately equal to distance between lateral ocellus and hind margin of vertex, and slightly longer than interocellar distance. Compound eyes convergent below, upper ocular distance 1.30 × lower ocular distance (Fig. 2). Hypostomal carinae subparallel, anterior angle acutely rounded (seemingly orthogonal in females). Antennal scape short, barely reaching midway between antennal socket and median ocellus; pedicel wider than long, two-thirds length of flagellomere 1; flagellomere 1 approximately half as long as flagellomere 2. Labrum short without apical process. Mandible simple, reaching opposing clypeal angle (Fig. 2). Pronotum maximum width 1.93 mm (1.86–2.03 mm); dorsolateral angle orthogonal as in the female (described as obtuse by Gibbs and Dumesh 2013); pronotal ridge uninterrupted carina mostly straight but with slight sinuation on lower half; pronotal lobe at most with a round projection (Fig. 1).

Inner metatibial spur absent. Tegula and forewing as in female except veins 1rs-m and 2rs-m not noticeably weak; hamuli arranged 4-1-3. Propodeum as in female. T1 width 0.71 × T2 width (Figs 3, 4). Hind leg noticeably slender, metafemur about six times as long as wide, metatibia about eight times as long as wide.

***Surface sculpture*.** Face as in female, except frontal areas somewhat more granular (Fig. 2). Lower part of gena and subgena striolate on oblique view [described as striolate in female by Gibbs and Dumesh 2013]. Mesoscutum and mesoscutellum similar to female, slightly more granular (Fig. 4). Mesepisternum coarsely granular-imbricate throughout, except shiny anteriorly contiguous to pronotal ventral extension. Metepisternum as in female, but transverse carinulae stronger. Propodeum noticeably striolate on entire basal area (Figs 3, 4). Terga surface sculpture as in female.

***Color*.** Body blackish brown, as described for the female in the original description, with the following remarks: apical half of clypeus bright yellow (Fig. 2), mandible bright yellow on outer surface except edges and basal outer interspace dark brown and apical tooth reddish amber. Posterior surface of entire flagellum and apex of flagellomere 11 amber. Tegula light brown to amber, translucid (Fig. 4). Legs proximally dark brown, lighter starting on distal third of femur and continuing on tibia, becoming yellow-amber on basitarsus and remaining tarsomeres, particularly on front and middle legs, pretarsal claws brown on distal half (Fig. 1). Forewing membrane hyaline, amber-ish, with green, yellow, and purple highlights; anterior margin infuscate, not as deeply as originally described for female; wing veins and pterostigma brown to amber-brown (Fig. 1). Terga blackish brown, with anterior margins (apical impressed areas) slightly reddish and lateral extensions translucid amber (Fig. 4); T1 with yellowish integument proximal to propodeum; sterna 1–4 brown to dark-brown with some underlying yellow-brown coloration basally and laterally, sterna 5–6 amber (Figs 3–5).

***Pubescence*.** As described for the female, with the following remarks: facial setae longer on frontal, antennal scape and lower gena (1.5–2.5 OD); tomentum dense on paraocular and supraclypeal areas, less dense but noticeable on upper clypeal section (Fig. 2). Mesepisternum and metepisternum with denser, shorter (as compared to female) subappressed setae; lower metepisternal and lateral surface of propodeum tomentose, posterior surface of propodeum densely tomentose. Coxae with long (1.0–1.5 OD) setae, otherwise legs with short, subappressed setae. T2–T4 with bands as in female; metasomal sterna with long (1.5–2.5 OD), subappressed setae, appearing simple, arranged in bands coming off gradulus.

***Genitalia*.** Sterna VII–VIII where not dissected. Genital capsule similar to that of L. (E.) nycteris, except as noted; gonobase short; gonocoxite noticeably strigulate, streaks not depicted in original description of L. (E.) nycteris; gonostylus moderately elongate, with two processes, trapezoidal apical process with scattered setae on its inner facing surface (a few long ones) appearing simple as opposed to the branched ones showed for L. (E.) nycteris in drawing by McGinley (1999), and retrorse membranous lobe about two-thirds length of gonocoxite, covered with minute moderately dense setae on its outer surface; volsella seemingly with prominent lateral lobe (Figs 6–8).

**Figure 9.**
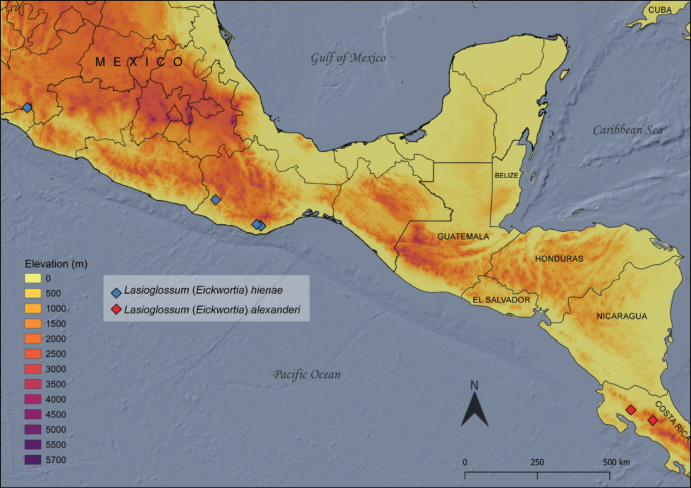
Known localities of Lasioglossum (Eickwortia) hienae and L. (E.) alexanderi.

**New distributional records.** The single female used for the original description of the species by Gibbs and Dumesh (2013) was collected from the southern slope of the Colima volcano, state of Colima in west-central Mexico. In this study we present specimens, including the three previously unknown males, that expand the distribution of L. (E.) hienae east and south within Mexico; all are from localities in the state of Oaxaca with elevations ranging from around 900 to 1730 m (Fig. 9).

### ﻿ Lasioglossum (Eickwortia) nycteris

Taxon classificationAnimaliaHymenopteraHalictidae

(Vachal)

F06949F4-2161-52E5-A90D-BBD2C92D2348

Halictus nycteris Vachal, 1904: 119 (♀).

#### Material examined.

Mexico • 2 ♂♂; Hidalgo; 38 mi. NE Jacala; 10 Jul 1961; 3100′; U. Kans. Mex. Exped. leg.; SEMC; 1 ♂; Hidalgo; 10 kme E Otanga; 10 Nov 1991; 1110 m; R. Ayala leg.; CNIN; 1 ♂; Hidalgo; Tenango de Doria, El Dano; 20.235, −98.227; 11 Mar 1993; 1680 m; L. Godinez leg.; CNIN;

### ﻿ Lasioglossum (Eickwortia) alexanderi

Taxon classificationAnimaliaHymenopteraHalictidae

(McGinley)

7A866588-07D6-5A03-A3CD-C634F010F5DB

Figs 10–15 , 16–18 , 19–21

Eickwortia alexanderi McGinley 1999: 118 (♀).

#### Material examined.

10 specimens (2 ♂♂, 8 ♀♀). Costa Rica • 2 ♂♂, 7 ♀♀; Puntarenas, Monteverde, 10.3181, −84.8025; 09 Feb2020; T. McMahon leg; on yellow composite;WRME; 1 ♀; Puntarenas; Monteverde: 20/24 Jun 1986; W. Hanson | G. Bohart leg.; BBSL.

#### Diagnosis.

The male of L. (E.) alexanderi is unique among all known Western Hemisphere *Lasioglossum* for the thickened apex of the clypeus above the labrum. The broad hind basitarsus is also unlike any *Lasioglossum* in the region. The female is entirely brown-black, mandibles bidentate, and wings entirely infuscate. The metapostnotum is crescent-shaped, defined apically by a sharp edge, and covered by subparallel. longitudinal carinulae (Fig. 21; McGinley 1999: fig. 13). An undescribed female collected simultaneously is remarkably similar, but the metapostnotum has a smooth apicolateral area, not sharply defined from the carinulate portion. This undescribed female is notably smaller, with varying levels of golden-green reflections and the apical terga are orange.

#### Description.

**Male**:

Length 8.3–9.2 mm.

Head length 2.4–2.5 mm. Head width 2.8–2.9 mm. Intertegular span 2.0–2.2 mm.

Forewing length 7.7–8.3 mm.

Maximum metasomal width (T3) 2.3–2.35 mm.

**Figures 10–15.**
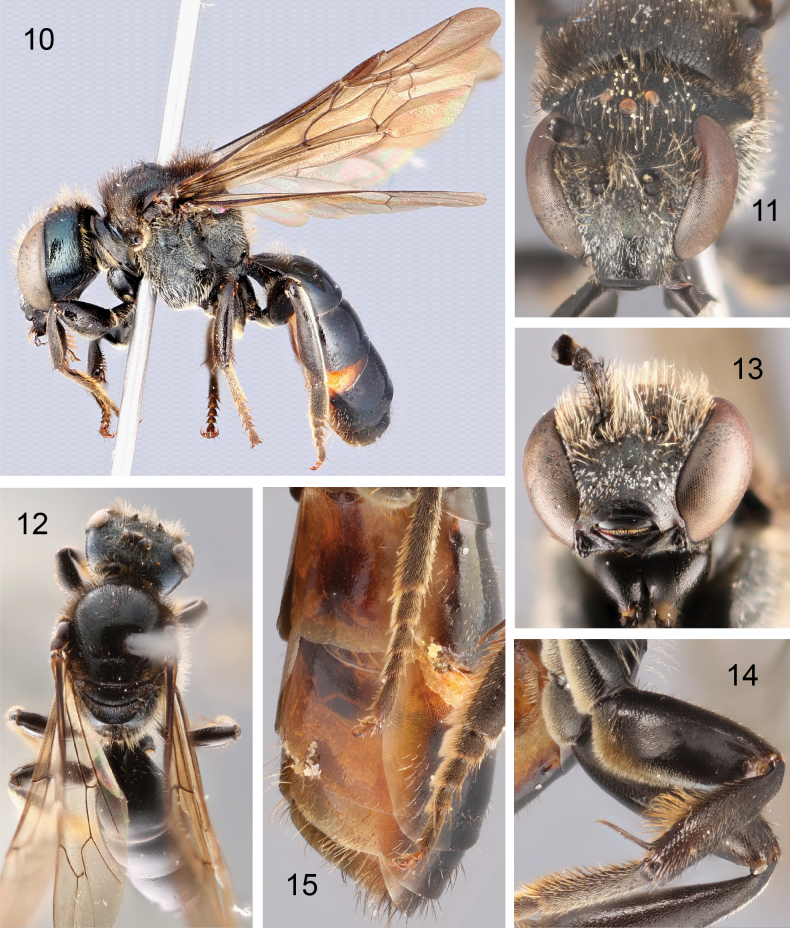
Male of Lasioglossum (Eickwortia) alexanderi**10** lateral habitus **11** face **12** dorsal habitus **13** mandibles and lower section of head **14** anterior surface of mesofemur **15** view of sterna.

***Structure*.** Head wider than long (length/width ratio = 0.85–0.91). Gena width at midlength 1.1–1.2 × width of contiguous section of compound eye in lateral view (Fig. 10). Supraclypeal area weakly protuberant. Clypeus width approximately twice length, width/length ratio = 2.2, projecting approximately half its length below lower margin of eyes (Fig. 11). Clypeal apex thickened, half-length of labrum (Fig. 13). Ocellocular distance 2.5 × lateral ocellar diameter, slightly greater than distance between lateral ocellus and hind margin of vertex, and 1.3 × interocellar distance (Fig. 12). Compound eyes convergent below, upper ocular distance 1.4 × lower ocular distance (Fig. 2). Hypostomal carinae strong, subparallel; anterior angle rounded. Antennal scape short, not reaching median ocellus; pedicel wider than long, three quarters length of flagellomere 1 (subsequent flagellomeres missing). Labrum short without apical process. Mandible simple, relatively broad at midlength, nearly reaching opposing clypeal angle (Fig. 13). Pronotum maximum width 2.7–2.8; dorsolateral angle orthogonal; pronotal ridge strongly angled, interrupted by oblique sulcus; pronotal lobe with anterior carina. Inner metatibial spur ciliate, subequal in size to outer metatibial spur. Tegula ovoid. Forewing veins 1rs-m and 2rs-m not noticeably weak; hamuli arranged 3-1-2.

**Figures 16–18.**
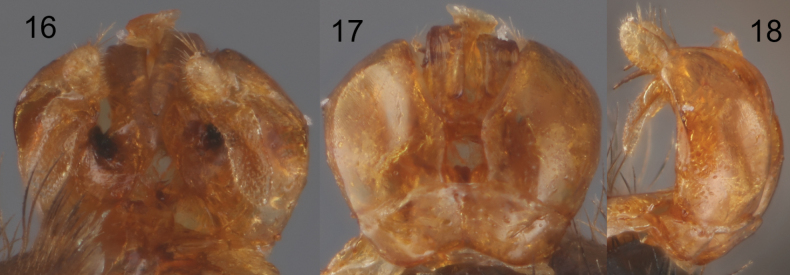
Male genitalic capsule of Lasioglossum (Eickwortia) alexanderi**16** ventral aspect **17** dorsal aspect **18** lateral aspect.

***Surface sculpture*.** Face tessellate-granular below antennal sockets, scabriculous above (Fig. 11), Gena and subgena striolate in oblique view, outer subgena polished. Mesoscutum and mesoscutellum polished, anteromedial mesoscutum imbricate, becoming striolate anterolaterally (Fig. 13). Metapostnotum with dense, regular carinulae, slightly depressed at apex (Fig. 13). Mesepisternum transversely striolate on upper half, vertically striolate on anterior lower half, more irregularly sculptured towards mesocoxa. Metepisternum transverse carinulae above, imbricate, irregularly sculptured below.

Propodeum lateral surfaces similar to lower metepisternum, posterior surface tessellate noticeably.

Terga polished.

***Color*.** Body blackish brown, with the following remarks: supraclypeal area with dull golden-green reflections, gena hints of green in certain lights, posterior surface of flagellomere 1 dark. Tegula dark brown (Fig. 12). Legs dark brown, pretarsal claws reddish brown (Fig. 15). Forewing membrane weakly infuscate throughout, with green and purple highlights; wing veins and pterostigma brown (Fig. 10). Metapostotum with blue tinge between carinulae. Terga blackish brown, with narrow rims reddish brown and ventrally reflexed portions amber; sterna amber (Fig. 15).

***Pubescence*.** Head and mesosoma with erect branched setae (1.5–2.5 OD); tomentum sparse on clypeus, paraocular and supraclypeal areas (Fig. 11). Pronotal collar, lobe and adjacent pre-episternum with tomentum. Meso- and metacoxae and trochanters ventrally with dense velvety setae, extending onto ventral surface of mesofemur (Fig. 14), otherwise legs with short subappressed setae. Apical half of pro- and mesotibiae with long setae ventrally, somewhat curved apically becoming straight towards apex. T1 anterior face with erect setae, remaining terga with sparse short setae, except longer on premarginal lines of T5–T6. S1–S4 nearly bare, S5–S6 with long setae.

***Genitalia*.** As in Figs 16–18. S7 with slender median process, S8 broadly rounded apically. Gonocoxite dorsally with inner edge subparallel for length approximately equal to gonobase (Fig. 17); gonostylus moderately elongate, rounded apically (Fig. 18); retrorse lobe present, moderately long, slender, rounded apically (Fig. 16); volsella with prominent lateral lobe.

**Figures 19–21.**
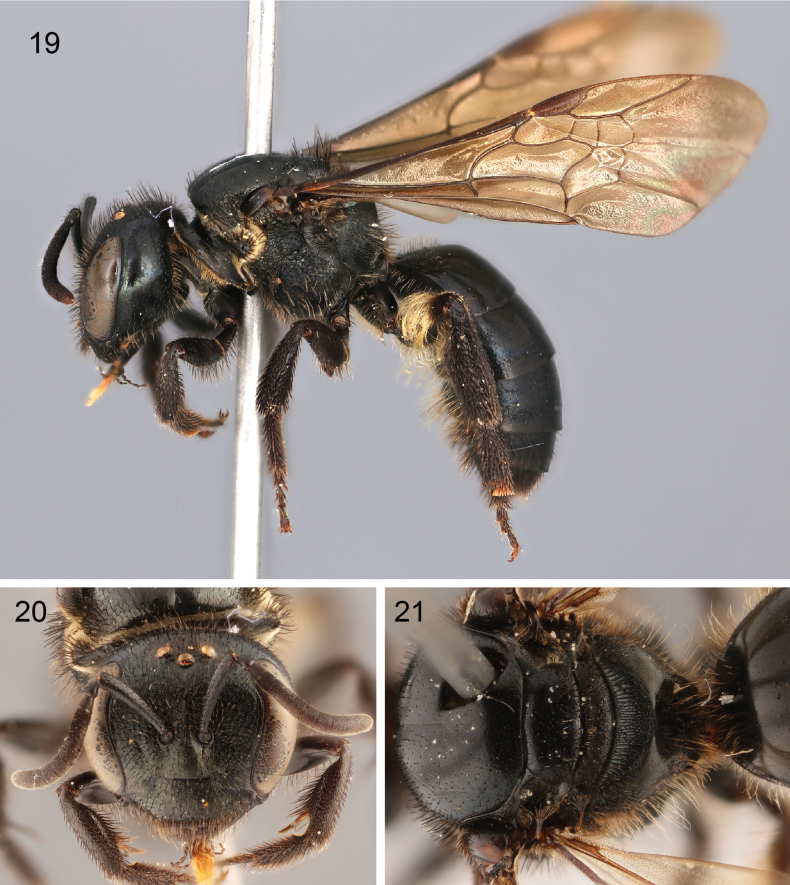
Female of Lasioglossum (Eickwortia) alexanderi**19** lateral habitus **20** face **21** dorsal view of mesosoma.

##### ﻿New distributional records

Although the holotype of *L.alexanderi* is lost, the description and figures are sufficient to recognize this unique species. At this point a neotype designation is not needed, and there is still hope the holotype may reemerge once more material in the former possession of McGinley is examined. The novelty of the male and co-occurrence with the female supports the association. The undescribed female co-occurring with *L.alexanderi* is unlikely to be the female based on the similar structure of the male and female metapostnotum, in addition to body color and size. The new collection site is approximately 70 km west of the type locality (Costa Rica; Heredia; 8.7 km N Varablanca).

### ﻿Revised key to Lasioglossum (Eickwortia) sensu stricto

Modified from Gibbs and Dumesh 2013. Characters to distinguish males of *L.hienae* and *L.nycteris* are not currently known.

**Table:** 

1	Both sexes: forewing complete infuscate; mesoscutum polished with subtle tessellation, distinct punctures; pronotal dorsolateral angle not carinate; metapostnotum distinctly carinulate. Males: posterior surface of propodeum without tomentum; T1 not slender; inner metatibial spur normal sized; hind basitarsus less than 3 × longer than wide	***L.alexanderi***
–	Both sexes: forewing anterior third infuscate; mesoscutum dull with dense, fine punctures and scattered coarser ones; pronotal dorsolateral angle carinate; metapostnotum smooth, with scarcely visible carinulae. Males: posterior surface of propodeum covered by tomentum; male T1 slender; inner metatibial spur short or absent; hind basitarsus greater than 6 × longer than wide	**2**
2	Female mesoscutum granulate, obscurely doubly punctate	***L.hienae***
–	Female mesoscutum tessellate with distinct double punctures (fine punctures separated by 1–2 diameters, coarse punctures separated by 5–10 diameters)	***L.nycteris***

## ﻿Discussion

*Lasioglossum* is by far the most diverse genus of bees (Ascher and Pickering 2024), with the number of species possibly reaching 2000 given the numerous undescribed species in several areas of the world. The mountainous regions of Mexico are a good example of areas where many *Lasioglossum* species remain unassigned to a known taxon (i.e. Fierros-López 1998; Hinojosa-Díaz 2003; Godínez-García et al. 2004; Bonet Ferrer and Vergara 2019; Razo-León et al. 2024). The present contribution adds to the knowledge of a characteristic group of species within *Lasioglossum*. The subgenus Eickwortia in its restricted sense includes only three very apomorphic species occurring in mountainous areas of Mexico and Central America, two of them known by a scarce number of female specimens. With the description of the males of L. (E.) hienae and L. (E.) alexanderi, we increase the knowledge on the morphological circumscription of this singular assemblage of species within the highly diverse *Lasioglossum*. Some of the features found in *Eickwortia* (*sensu stricto*) (i.e. apically bidentate mandibles of females), as traditionally understood, are also present in some Neotropical *Lasioglossum* recently found to be phylogenetically closely related (Gardner 2023), which leads to the possibility of reconsideration of the taxonomic definition of the group.

The male of *L.alexanderi* has unique features but lacks the autapomorphic characters of *L.nycteris* and *L.hienae*. However, its clypeus and hind leg characters are unlike other *Lasioglossum* known to us. In some ways, *L.alexanderi* seems intermediate between *L.nycteris* and the apparently related metallic species. This is further highlighted by the undescribed female found, which is so similar to *L.alexanderi* that a plausible hypothesis would be that it is a caste of the same species, with associated structural and color differences. Under certain lighting, *L.alexanderi* shows signs of metallic reflections on the gena and metapostnotum. Future study of this broader definition of *Eickwortia* is needed to contextualize the patterns seen in the females. Males of the metallic species share some similarities to *Eickwortia* (*sensu stricto*), but they seem to have fewer evident unique characters than the three species traditionally included.

In terms of distribution, most of the 30 L. (E.) hienae specimens here studied expand the known range of the species east, along the Sierra Madre del Sur range in the state of Oaxaca, Mexico. While the type locality given by Gibbs and Dumesh (2013) set the species in the mountains of the Transmexican volcanic belt. These two mountainous assemblages are biogeographic provinces of a major biogeographical assemblage, the Mexican transition zone, an area characterized by a mix of taxa with affinities to the Nearctic and Neotropical regions (Morrone 2019). For this region a few distributional patterns have been recognized (Morrone 2019), depending on the affinities of the species assemblages found in them. The series of L. (E.) alexanderi examined here were recorded at elevations above 1500 m, as was the holotype. *Eickwortia*, either in its current sense or in an expanded redefinition, seems to be allied to high elevations in the area, but more distributional records are needed for the included species.

Males of L. (E.) nycteris currently held at WRME (in the material examined above, referred to as deposited at CNIN) were compared to L. (E.) hienae males using photographs and discussion between the co-authors with reference to the detailed description of McGinley (1999). Available male L. (E.) nycteris have had the genitalia removed, but they are not associated with the specimens. Our statements of the seemingly lack of morphological differentiation of our newly described L. (E.) hienae males with those of L. (E.) nycteris are based on the comparison with the detailed original description of this (McGinley 1999). The newly described males were collected during the same collecting events as the females morphologically matching L. (E.) hienae, while no specimen of L. (E.) nycteris is known from those events.

The allometric variability observed in females of L. (E.) nycteris is also seen in the several females revised for this study. However, details of such variation are to be examined in future studies by the authors. The strongly bidentate mandible has been speculated to be an adaptation for nesting in wood, although given the moderate degree of macrocephaly it could potentially be related to intraspecific interactions. There is no evidence of nesting biology for these species; however, the *Hemihalictus* series displays a wide range of nesting behaviour (Michener 1974). *Lasioglossumfigueresi* Wcislo is closely related and shows both solitary nesting and some low-level social nesting (Wcislo et al. 1993). A lot of work remains to be done to understand the biology and taxonomy of Lasioglossum (Eickwortia), but with this study we hope to contribute to a better understanding of the behavior, morphology, and distribution of the group.

## Supplementary Material

XML Treatment for Lasioglossum (Eickwortia) hienae

XML Treatment for Lasioglossum (Eickwortia) nycteris

XML Treatment for Lasioglossum (Eickwortia) alexanderi

## ﻿Additional information

### Author ORCIDs

Ismael A. Hinojosa-Díaz https://orcid.org/0000-0002-5493-850X

Ana Celeste Martínez Cervantes https://orcid.org/0000-0001-5707-1051

Jason Gibbs https://orcid.org/0000-0002-4945-5423

### Data availability

All of the data that support the findings of this study are available in the main text.
